# Supplementary material for: Are carbon emissions trading and green financial instruments synergistic? -Comprehensive quantitative research based on content analysis
Source: PLoS One. 2024 Mar 7;19(3):e0298601. doi: 10.1371/journal.pone.0298601 (PMC10919601; doi:10.1371/journal.pone.0298601)
Supplement: S1 Data — (ZIP) [file pone.0298601.s001.zip › Data/地级市Z_F工作报告.zip!/║╙─╧/┐¬╖Γ╩╨/└┤╫╘╣1⁄2╓┌║┼í╛┬φ┐╦╩2╛▌═°í┐_2012.docx]

各位代表：

现在，我代表市人民政府，向大会作政府工作报告，请予审议，并请各位政协委员和列席人员提出意见。

一、2011年工作回顾

2011年，是开封发展历程中很不平凡的一年。一年来，在省委、省政府和市委的正确领导下，在市人大、市政协的监督支持下，全市上下深入贯彻落实科学发展观，坚持“四个重在”实践要领，紧紧围绕市十次党代会确定的打好“五大攻坚战”、实现“五个翻番”的工作目标，抢抓中原经济区建设、郑汴一体化发展等重大机遇，凝心聚力抓发展，金石开封谋跨越，较好地完成了市十三届人大三次会议确定的各项任务。全市生产总值1093.6亿元，增长12.9%；城镇固定资产投资584.8亿元，增长33.1%；社会消费品零售总额434.6亿元，增长19.2%；规模以上工业增加值355亿元，增长23.6%；财政一般预算收入49亿元，增长32.5%，其中市本级一般预算收入突破20亿元。城镇固定资产投资、社会消费品零售总额、财政一般预算收入增幅均居全省第1位，规模以上工业增加值增幅居全省第3位。经济社会发展继续保持好的趋势、好的态势和好的气势，在“十二五”开局之年成功迈上“千亿GDP”新征程，为圆满完成市十次党代会确定的奋斗目标，在中原经济区建设中快速崛起奠定了坚实基础。

（一）工业经济实现新跨越。重点企业培育成效显著。全市规模以上工业企业达到1080家，主营业务收入超亿元企业达到296家，“双30”企业主营业务收入增长35%以上，开封空分、新大新等24家企业主营业务收入超10亿元，晋开集团综合收入超过40亿元，被省政府评为“河南省百强企业”。工业投资力度持续加大。奇瑞汽车（河南）制造基地、晋开集团百万吨总氨、龙宇化工聚甲醛二期、河南百昱新型平板显示电容式触摸屏等144个重点工业项目顺利实施，完成工业投资246.8亿元，增长45.4%。产业集聚区建设明显加快。全市8个产业集聚区完成固定资产投资354.2亿元，增长69.4%。十大产业集群规模以上工业企业达到879家，累计实现主营业务收入1178亿元，增长40.4%。重点项目建设实现新突破。强力实施转型升级“1538”工程，深入开展重大项目百日推进活动，全市304个重点项目完成投资444.6亿元，增长39.6%，带动全社会固定资产投资突破600亿元，是历史上投资规模、投资强度最大的一年。

（二）“三农”工作取得新进展。农业综合生产能力进一步增强。强力推进粮食核心区建设，全市粮食总产259万吨，增长1.3%，连续8年创历史新高。认真落实国家强农惠农富农政策，各项财政惠农补贴9.8亿元。农业产业化进程不断加快。新认定无公害农产品生产基地14个、无公害农产品31个，省级农业产业化龙头企业增加到15家，农民专业合作社发展到1696家，农业机械化率达到76.6%。全市标准化养殖小区达到486个，规模养殖场（户）超过4万个。新型农村社区建设扎实推进。完成91个新型农村社区建设规划，启动35个新型农村社区建设。完善农村基础设施，新建沼气池6899座，改建农村卫生厕所12240座，新解决22.3万农村群众饮水安全问题。完成扶贫开发整村推进村41个，5.6万农村贫困人口实现脱贫。

（三）第三产业发展有了新成效。文化产业园区建设加速推进。开封宋都古城文化产业园区成功升格为国家级文化产业示范园区。完成书店街特色街区改造，刘青霞故居纪念馆建成开放，城墙保护四期工程基本完成，繁塔·禹王台景区开发、大宋文化博物馆·开封城摞城新郑门遗址等一批重点文化旅游项目扎实推进。重大文化旅游活动顺利开展。成功举办中国（开封）2011清明文化节、中国开封第29届菊花花会暨第三届中国收藏文化（开封）论坛等重大文化旅游活动，节会经济成效显著。旅游业发展迅速。持续提升景区品位，不断加大旅游推介力度，全市旅游接待量3924万人次，增长13.2%，旅游综合收入达到155亿元，增长21%。金融业健康发展。全市金融机构各项存款余额787.6亿元、贷款余额443.8亿元，新增存贷款104.5亿元和44.8亿元，分别较年初增长15.3%和11.2%。澳洲联邦银行（兰考）村镇银行、开封新东方村镇银行建成开业。服务业快速发展。大润发综合购物广场、大商集团新玛特建成开业，商贸、住宿、餐饮发展层次进一步提升，邮政、电信、交通运输和房地产业健康发展。全市第三产业增加值增长13.2%。

（四）开封新区建设迈出新步伐。综合规划进一步完善。完成了运粮河组团城市设计。完善了启动区控制性详规、铁南基础设施规划以及新区道路管网修建性详规。《开封新区产业发展总体规划》终期成果通过专家评审。基础设施建设不断加快。新区一期、二期工程18条道路全部建成通车，三期、四期基础设施建设进展顺利，中心区和铁南工业区基础设施BT项目扎实推进，建成及在建道路110公里，1—12大街交通网络基本形成。重点项目建设成效显著。总投资446.3亿元的74个重大项目完成年度投资82亿元。汽车及零部件产业园、大型空分产业园等初具规模。黑岗口调蓄水库开工建设。迁村并居扎实推进。职教园区建设进展顺利，省化工技校、省医药技校入驻新校区。主导产业培育力度加大。深化专业园区建设，入驻企业达到486家，初步形成汽车及零部件、装备制造、食品及农副产品深加工等三大主导产业集群。汴西产业集聚区被命名为“河南省创新型产业集聚区”。

（五）城乡规划建设管理水平得到新提升。城乡规划编制成效显著。编制了《开封市城市近期建设规划（2011—2015）》和古城区、东城区、南城区、西城区控制性详规。完成了五县县域村镇体系规划、五区村庄布局规划和15个中心镇核心控制区控制性详规。城乡基础设施建设不断加强。郑民高速一期建成通车，郑汴物流通道开封段基本完工，郑开城际铁路、连霍高速公路拓宽改造、郑徐客运专线建设稳步推进。完成了宋城路、大庆路、魏都路景观整治；南干渠、东护城河清淤以及东区沉陷点维修工程顺利完工；东陈庄路、万兴园路、内顺城路改造、东外环路北延、电视塔南路工程正式启动。完成人防工程建设12.6万平方米。新建改建县乡公路227.2公里，改造大中型危桥880延米。城市管理取得新突破。数字化城市管理系统正式建成投用，解决城市管理案件18万件，一大批涉及城市管理和市民生活的问题得到及时有效解决。依法整治规范市区三轮车工作取得显著成果，取缔非法客运三轮车8488辆；投入6500万元，更新城市公交车254辆，新开辟调整公交线路16条，城市交通秩序明显好转。集中开展市区墙体整治和违法建设整治专项活动，加强环境卫生管理，市容市貌进一步改观。城镇化步伐不断加快。以各县产业集聚区为载体，不断加大基础设施建设力度，促进产城融合，拉大县城框架，吸引农村人口向县城和小城镇聚集。

（六）改革开放取得新突破。企业改革不断深化。开封塑料厂、丝织印染厂进入破产清算阶段；开化集团、中州机械厂、缝纫机总厂、针织厂等企业破产清算工作基本结束。社会事业改革扎实推进。基层医疗卫生机构综合改革进展顺利，93个乡镇卫生院、市区7个社区卫生服务中心及所有村级卫生室实现基本药品零差率销售；事业单位岗位设置管理工作如期完成；城乡居民社会养老保险试点工作继续深化。警务机制改革深入推进。对外开放工作成绩显著。加强与国内外500强、央企、外资企业合作，全市新签约亿元以上项目162个，总投资1037.6亿元。实际利用外资2.3亿美元，增长82%；外贸出口2.3亿美元，增长21%；引进省外资金261.7亿元，增长47.4%。我市被省政府评为“对外开放工作优秀省辖市”。

（七）发展方式有了新转变。加快构建自主创新体系。全市国家高新技术企业达到11家，高新技术产业实现产值100亿元。实施新开电器铁路电力远动智能箱式变电站等33项省级科技计划，河南省化学原料药制备、糖精工程、米面制品工程技术研究中心被认定为省级工程技术研究中心。加强与中国农业大学等高等院校合作共建，开展技术合作项目30个。河南大学棉花生物学国家重点实验室获准组建，实现省高校国家重点实验室“零”的突破。我市被评为2011年全国科技进步先进市。扎实推进节能减排。实施建筑节能、公共机构节能等八大节能工程。全面完成省定淘汰落后产能任务。提升污水处理厂配套管网建设，加强重点排污企业监管，全面完成减排任务。加强环境保护和生态建设。强化主要污染物排放总量控制，全市出境河流断面水质考核均达到省定目标，饮用水源地水质平均达标率保持100%。全年空气环境质量优良天数达到323天。加强土地资源管理调控，连续12年实现耕地占补平衡。继续推进林业生态市建设，完成造林面积17.2万亩。

（八）民生幸福指数实现新提高。从解决人民群众最关心、最直接、最现实的利益问题入手，落实资金35亿元，圆满完成十五项民生工程。2011年，全市城镇居民人均可支配收入15558元，增长13.6%；农民人均纯收入6492元，增长20.4%。就业和再就业工作不断加强。全市城镇新增就业8.8万人，失业人员再就业4万人，帮助困难人员就业1.8万人，动态消除“零就业家庭”，城镇登记失业率控制在3.85%。农村劳动力转移就业94.6万人，实现劳务收入108.6亿元。社会保障水平进一步提高。市区、各县最低工资标准分别提高到950元、820元。城乡低保标准和农村五保年分散供养、集中供养标准进一步提高，基本实现应保尽保。为16.3万名企业退休人员人均月增加养老金196.3元。为37.4万名60岁以上城乡居民发放养老保险金1.7亿元。城镇基本医疗保险、生育保险实现市级统筹。社会福利园区建设项目加快推进。保障性安居工程建设力度加大。开工建设经济适用住房、廉租住房、公共租赁住房等各类保障性住房17353套，开工率100%。为1.7万户城市低收入住房困难家庭累计发放租赁补贴6178万元。全市归集住房公积金4.7亿元，发放个人住房公积金贷款2.5亿元。医疗卫生条件持续改善。全市城乡居民人均基本公共卫生服务经费标准提高到25元。新型农村合作医疗财政补助标准提高到200元、大病住院报销封顶线提高到10万元，参合率达到97.6%以上。完成603个行政村卫生室建设，新建改建社区卫生服务中心7个。教育体育事业加快发展。实施学前教育三年行动计划，大力推进义务教育均衡发展，新改扩建幼儿园105所，发放农村义务教育经费5.2亿元。累计投入资金6.2亿元，基本完成中小学校舍安全工程三年攻坚目标任务。成功举办2011中国郑开国际马拉松赛和开封市第八届运动会。和谐社会建设成效显著。切实加强安全生产监管，继续开展食品药品安全专项整治，全市没有发生重大安全生产和食品药品安全事故。扎实推进平安开封建设，狠抓社会治安综合治理，严厉打击各种违法犯罪，切实加强信访稳定工作，社会大局保持和谐稳定。

过去的一年，我们坚持不懈强化政府自身建设，认真落实“三具两基一抓手”工作法，持续推进“两转两提”，以领导方式转变加快发展方式转变，各级政府机关服务效能和工作水平进一步提升。坚持依法行政，自觉接受人大及其常委会监督，支持政协履行职能，认真办理人大代表议案、建议和政协提案。积极推进政府廉政建设，强化财政监察审计监督。加强精神文明建设，全市文明素质有了较大提高。大力开展国防教育，扎实做好“双拥”工作，第6次荣获“全国双拥模范城”荣誉称号，驻汴解放军和武警部队在支持地方经济建设、维护社会稳定等方面做出了重要贡献。切实加强民族宗教工作，各民族共同团结进步、共同繁荣发展的局面进一步巩固。外事、侨务、对台、统计、档案、史志、地震、气象、社会科学等工作都取得了新的成绩。

各位代表：

过去的一年，是全市上下在复杂多变的环境中迎挑战、谋崛起的一年，是“十二五”开局之年抢机遇、求跨越的一年，是经济社会发展再上新台阶、取得新突破的一年。这些成绩的取得，是省委、省政府和市委正确领导的结果，是市人大、市政协监督、支持的结果，是全市人民齐心协力、努力拼搏的结果。在此，我代表市人民政府，向工作在全市各条战线上的工人、农民、知识分子、干部，向驻汴解放军指战员、武警官兵和公安民警，向各民主党派、工商联及社会各界人士致以崇高的敬意！向所有关心、支持开封发展的同志们、朋友们表示衷心的感谢！

各位代表！

在肯定成绩的同时，我们清醒地认识到，开封仍处于工业化、城镇化、农业产业化的进程之中，仍处于结构调整优化、发展方式转变的进程之中，仍处于蓄势积累、快速崛起的进程之中。我市经济社会发展中还存在不少困难和问题，主要表现在：经济总量小、底子薄的局面还没有从根本上得到改变；经济结构性矛盾仍较突出，加快经济结构调整、转变经济发展方式的任务还十分繁重；资金、土地、资源环境等制约因素突出，经济发展瓶颈亟待破解；城市基础设施欠账较多，县域经济实力不强，农村公共服务和基础设施仍较薄弱，统筹城乡发展任重道远；财政保障能力不足，支出压力加大，社会事业发展依然滞后，一些涉及群众切身利益的问题还没有解决好；一些部门职能和作风转变还不到位，少数干部以权谋私、为政不廉的现象仍然存在，政府自身建设仍需继续加强，等等。对此，我们一定高度重视、认真加以解决。

二、2012年工作的总体要求和任务

今年是 “十二五”时期承先启后的重要一年，是推动开封快速崛起的重要一年，我们要恪尽职守、攻坚克难、锐意进取，向全市人民交一份满意的答卷。

当前，国内外经济形势复杂严峻，世界经济复苏进程艰难曲折，国内区域竞争更为激烈，我市经济发展中不平衡、不协调、不可持续的矛盾和问题仍很突出，保持经济社会发展良好态势面临诸多挑战。但总的来看，开封正处于风生水起的黄金发展期，发展优势明显，发展机遇难得。国务院出台的《关于支持河南省加快建设中原经济区的指导意见》，明确提出要“支持郑汴新区加快发展，建设内陆开发开放高地，打造‘三化’协调发展先导区，形成中原经济区最具活力的发展区域”，省九次党代会进一步提出要“推进郑汴一体化发展，促进郑州、开封融合发展，努力打造中原经济区核心增长板块”，进一步强化了开封作为中原经济区核心区的战略核心地位。党的十七届六中全会作出了推动社会主义文化大发展大繁荣的战略部署，开封宋都古城文化产业园区被命名为国家级文化产业示范园区，为建设国际文化旅游名城带来了新的机遇。市十次党代会明确提出打好“五大攻坚战”，实现“五个翻番”，促进开封快速崛起，描绘了未来五年开封发展的蓝图。特别是经过全市人民多年的不懈奋斗，我市经济发展速度连续四年超过全省平均水平，经济实力显著增强，全市上下思发展、谋发展、促发展的浓厚氛围已经形成。当前，开封发展承天时、秉地利、聚人和，扬帆破浪风正劲，快速崛起正当时。我们既要保持清醒忧患意识，善于趋利避害，更要牢牢把握重要战略机遇期，坚定必胜信心、乘势而上，在快速崛起的道路上迈出更快、更大的步伐。

今年政府工作的总体要求是：坚持以邓小平理论和“三个代表”重要思想为指导，深入贯彻落实科学发展观，坚持“四个重在”实践要领，按照市十次党代会作出的战略部署，抢抓中原经济区建设和郑汴一体化发展等重大机遇，围绕中原经济区核心区、“三化”协调发展先导区、文化产业发展示范区、新型工业化城市和国际文化旅游名城“五个定位”，全力打好工业强市、开封新区五年成规模、建设国际文化旅游名城、推进新型城镇化、农业产业化提升“五大攻坚战”，持续求快，务实发展，积极作为，不断壮大综合实力，不断提升城市品位，不断提高人民群众幸福指数，为实现开封快速崛起不懈奋斗，以优异成绩迎接党的十八大胜利召开。

今年全市经济社会发展的主要预期目标是：生产总值增长12%；公共财政预算收入增长15%以上；规模以上工业增加值增长20%；全社会固定资产投资增长23%；社会消费品零售总额增长18%；外贸出口增长15%；实际利用外资增长20%；居民消费价格涨幅控制在4%左右；城镇居民人均可支配收入增长10%左右；农民人均纯收入增长10%左右；人口自然增长率控制在6.5‰以内；城镇新增就业5.8万人，城镇登记失业率控制在4.5%以内；节能减排完成省下达任务。

实现上述要求和目标，做好今年工作，必须按照中央“稳中求进”和省委“持续求进”要求，立足开封实际，把握“持续求快”的“总基调”，坚持“五个着力”，强力推进“五大攻坚战”。把握 “一个总基调”：“持续求快”作为今年我市经济工作的总基调，符合中央、省委精神，符合开封实际，符合群众期盼，是加快中原经济区建设、推进郑汴一体化发展的需要，是打好“五大攻坚战”、实现“五个翻番”、促进开封快速崛起的需要，更是惠民生、谋发展、破瓶颈、解难题的需要。“持续”就是保持开封科学发展好的思路、好的举措、好的势头，保持社会大局和谐稳定的好局面；“求快”就是在发展速度晋位升级上求快，在转变经济发展方式上求快，在深化改革开放上求快，在保障改善民生上求快。坚持“五个着力”：就是着力推进郑汴一体化发展，加快推进电信、金融、交通、产业、生态同城和资源共享；着力培育主导产业和骨干企业，增强全市经济发展后劲；着力破解资金、土地、资源环境等发展瓶颈，增强开封发展活力；着力优化经济发展环境，努力开创对外开放新局面；着力保障和改善民生，办好一批实实在在的民生工程。强力推进“五大攻坚战”：就是强力推进工业强市攻坚战，加快新型工业化步伐；强力推进开封新区五年成规模攻坚战，打造经济社会发展核心增长极；强力推进建设国际文化旅游名城攻坚战，提升发展软实力；强力推进新型城镇化攻坚战，全面提高城镇化水平；强力推进农业产业化提升攻坚战，夯实经济发展基础。

三、2012年重点工作

今年要重点做好以下九个方面的工作：

（一）抢抓中原经济区建设机遇，深入推进郑汴一体化发展。要牢牢抓住中原经济区建设难得机遇，主动对接，强力运作，推动郑汴一体化发展取得实质性进展。加快推进电信同城。积极做好开封电话号码升位和两市统一区号、并网等相关工作。加快推进金融同城。抓好郑汴资金清算同城化、票据融资同城化，加快两地资金周转速度，实现两地居民在各商业银行和股份制银行办理金融业务享受同城待遇。加快推进交通同城。扎实推进郑汴物流通道、连霍高速开封段扩宽、郑开城际铁路、郑徐客运专线等道路建设，启动310国道开封境汪屯至中牟界等新建改建工程，争取尉氏县至郑州航空物流港区通道开工建设。深入推进郑汴公交同城。加快推进产业同城。进一步增强两市在产业链、技术链、产品链、科技链方面的衔接与交流，着力推进两市产业互补、资源共享、功能协调发展。积极培育战略新兴产业，重点抓好汽车及零部件、富士康电子信息、港区物流等重大项目对接。加快推进生态同城。尽快启动郑汴新区水系建设，加快沿黄休闲生态旅游区开发。积极推进文化、教育、卫生、体育、科技、人才等各方面的对接和资源共享。

（二）强力推进工业强市攻坚战，加快新型工业化步伐。坚持把推进新型工业化作为快速崛起的关键，增加总量，提升质量，提高效益，不断增强工业实力。强力推进重点项目建设。开展重大项目建设攻坚行动，强力实施 “3536”工程，组织实施300个以上重大项目，完成投资500亿元以上，加快推进3大类60个重大项目建设。全力推进汽车零部件产业园、北大科技产业园、晋开集团百万吨总氨、中平能化光气产业园、盛世中原家居产业园等30个重大工业项目实施，确保重点工业项目投资完成228亿元。大力培育重点企业。完善企业服务长效机制，加大重点企业扶持力度，提升“双30”企业带动引领作用，力争销售收入超10亿元企业达到25家以上，超20亿元企业达到4家。加快平原非标、禾丰牧业、得胜锅炉等企业上市融资，推进企业债券发行工作。不断提升产业集聚区建设水平。加快产业集聚区基础设施建设，完成各产业集聚区起步区和发展区道路、供电等基础设施，实现基础设施全覆盖、与区外设施互联互通。突出抓好标准化厂房、检测检验、公共租赁住房、综合服务中心等生产生活公共服务设施建设，着力提升产业集聚区承载力。全面落实产业发展政策，加大招商引资力度，积极承接产业转移，加快汽车及零部件、装备、化工、新材料、食品、纺织服装、光伏、木业、生物医药、电子信息等十大产业集群发展，力争十大产业集群主营业务收入达到1300亿元，增长18%。加快完善工业服务体系建设。健全金融服务体系，强化银企对接，力争年内新增融资100亿元以上；加大政策扶持力度，大力发展民营经济，促进中小企业健康快速发展；加快现代物流、人才培养等公共服务体系建设，积极构建创业、研发、孵化中心等服务平台；实施信息化和工业化“两化融合”实验区示范工程，创建国家、省级新型工业化产业示范基地；抓好工业经济运行，加强煤电油气运供需调度，确保企业正常生产需要。

（三）强力推进开封新区五年成规模攻坚战，打造经济社会发展核心增长极。按照“呼应郑州，借力省会，保持好势头，实现新跨越”总要求，围绕“一年一个样、三年大变样、五年成规模”总目标，抢抓机遇，奋力跨越。加快基础设施建设。积极运作，开辟多元化融资渠道，加快完善启动区、铁南工业区基础设施，启动运粮河组团开发，力争完成基础设施投资50亿元。全面推进黑岗口调蓄水库及沿湖绿化、亮化、景观带建设，启动中心商务区建设，搞好金明大道提升改造，建成一批特色彰显、魅力独特的城市亮点。加快迁村并居步伐，完成闫砦、毛砦迁村并居一期工程，瞿家砦、野场55栋楼房竣工验收，启动王府砦迁村并居工程，扎实做好横堤铺、斗门等迁村并居前期工作。积极推进职业教育园区、开封高中西校区、市中心医院、解放军第155医院等配套服务设施建设，启动市体育场、规划馆、博物馆、科技馆、图书馆规划设计及建设，建成一批标准化厂房，增强新区吸纳能力。抓好重大项目建设。完善服务措施，落实跟踪机制，千方百计加快推进奇瑞汽车制造基地、河南煤化重型设备制造、银基数字科技研发、晋开总部、日本住友、国际金融中心等重大项目建设，确保完成固定资产投资200亿元以上。紧盯国内外500强企业，大力引进战略投资者，确保引进开工一批带动能力强、科技含量高的重大项目，力争一批跨国公司区域性总部、国内大型企业集团总部入驻。着力培育主导产业。加快汽车零部件产业园发展，建成中原经济区重要的汽车及零部件产业基地。推进国家级空分特色产业基地建设，提高空分产业核心竞争力。加快家具产业园建设，打造集家具生产、研发、交易于一体的家具产业集群。大力培育电子信息、新材料、新能源和生物医药等新兴产业，培育引进国家级科技创新型企业10家以上，建设高新技术产业化基地。大力发展现代服务业，重点发展金融业、商贸业和文化创意产业，建设区域性金融、商贸流通和科教研发中心。

（四）强力推进建设国际文化旅游名城攻坚战，提升发展软实力。认真落实党的十七届六中全会、省委九届二次全会精神，立足国际性、唯一性、时代性，强化城市建设精品意识，完善城市功能，提升开封美誉度，着力建设国际文化旅游名城。大力推进文化产业园区建设。认真落实《加快推进宋都古城文化产业园区建设的实施意见》，健全组织机构，完善发展规划，以宋文化为主题，以项目建设为载体，围绕宋文化集中展示区、华夏历史文明传承创新先行示范区、国际文化旅游名城总体定位，做强文化旅游、书画工艺美术、饮食文化、文艺演出、休闲娱乐、会展收藏、传媒出版、文化培训八大产业，推动文商旅融合发展，着力打造全城一景的特色文化产业基地。加强文化旅游项目谋划和建设。引进战略合作伙伴，整合文化旅游资源，加快宋都水系、鼓楼复建、繁塔·禹王台景区开发、延庆观扩建、大宋文化博物馆·开封城摞城新郑门遗址、朱仙镇文化生态旅游产业园等重点文化旅游项目建设。实施开封城墙保护五期工程。提升文化旅游节会活动品位。办好“两节会一论坛一赛事”及第22届中国厨师节等重大节会活动，继续抓好大型实景演出《大宋·东京梦华》等知名文化品牌建设。全面推进旅游标准化和信息化，加快构建与国际接轨的旅游服务体系，抓好市旅游集散综合服务中心等配套设施建设，提高旅游接待水平和服务质量，大力开拓文化旅游市场，确保全市旅游综合收入增长15%以上。大力发展服务业。加快中心商务功能区和特色商业区建设。加快香港爪哇大型休闲购物中心、丹尼斯百货、金明池商业广场等商务、休闲购物服务中心项目建设，启动养老产业园区建设，推动娱乐、休闲、酒店、健身、养老、会展等第三产业发展。围绕名店、名吃、名品、名厨，做精做实餐饮服务业。鼓励发展金融保险服务业，支持交通银行等更多股份制银行在开封设立分支机构。提升城市精细化管理水平。充实完善数字化城市管理系统内容，提升数字化城市管理系统运行质量。加强城市交通整治，巩固整治规范市区三轮车工作成果，提升出租车行业服务水平，优先发展公共交通，更新公交车100辆。大力开展“三城联创”活动，突出抓好市容环境、市政设施、园林绿化等方面的精细化管理，着力提高市民文明素质，把我们的城市建设得更美、更绿、更靓、更干净、更文明。

（五）强力推进新型城镇化攻坚战，全面提高城镇化水平。坚持以新型城镇化引领“三化”协调发展，以中心城区为核心，以县城为重要支撑，以中心镇为重要支点，以新型农村社区为重要依托，大力推进城乡一体化建设，着力构建“中心城区—县城—中心镇—社区”城镇化格局。下大力气抓好老城区改造。在强力推进开封新区建设的同时，下大决心抓好老城区改造，着力形成中心城区内老城区、新区、运粮河、东区四个板块协调发展的城市格局。大力度推进“两改一建”，即推进老城区棚户区、城中村改造和保障性安居工程建设。坚持政府主导与市场运作相结合、统筹规划与分步实施相结合、棚户区改造与提升文化产业功能相结合、原地安置与异地安置相结合、以人为本与依法征收相结合，鼓励成片成区域开发，鼓励文化旅游商贸类建设项目，鼓励城墙内机关、企业、居民外迁。城墙内严格控制建筑风格及建筑高度，严格控制局部零星开发，严格控制纯房地产开发项目。痛下决心，广泛动员，区为主体，部门联动，分步实施，和谐推进，到“十二五”末基本完成老城区棚户区改造任务。今年要实施棚户区、城中村改造303万平方米，开工建设经济适用住房2700套，廉租住房2600套，公共租赁住房3426套，棚户区改造安置住房2368套，着力缓解中低收入群体住房困难。抓好河南大学周边景观整治及水系二期周边地块开发。搞好环城景观带、城市出入口景观带建设，启动火车站及周边改造开发。强力实施新曹路（护城堤—大广高速）改造、夷山大街北延和南延、西环路北延、劳动路北延、东京大道东延、黄河大街南延工程，打通迎宾路北段。扎实推进生活垃圾中转站、旱厕改造、华北体育场改造、东护城河整治等基础设施项目建设，继续改造升级农贸市场和夜市。优化服务设施布局，进一步完善城市功能。加快县城和小城镇建设步伐。深入实施城乡建设三年大提升计划，投资100亿元，全面推进城镇基础设施建设。坚持用现代城市的理念和标准规划建设县城，突出县域特色，强化产业支撑，创造更多的就业岗位。积极推进户籍制度改革，放宽落户条件，促进农民向县城、中心镇转移。加快城乡交通基础设施建设，升级改造国省干线公路。加快县城城中村改造步伐。把小城镇建设作为统筹城乡发展的重要节点，促进基础设施向农村延伸、公共服务向农民覆盖，加快朱仙镇等中心镇开发建设。扎实推进新型农村社区建设。按照“科学规划、适度超前，先易后难、梯次推进，尊重民意、因村设计，市场运作、多元投入”的原则，稳步推进新型农村社区建设。加快规划编制，高标准编制新型农村社区修建性详规。加大资金投入，整合市县区资金，加快已启动35个新型农村社区基础设施和公共服务配套设施建设。今年每个县区至少建成1个1000户以上的新型农村社区，各乡镇（涉农办事处）年底前至少开工建设1个新型农村社区。注重村庄布局与发展当地主导特色产业相结合，吸引社会资本投入农村，大力发展二、三产业。

（六）强力推进农业产业化提升攻坚战，夯实经济发展基础。坚持把新型农业产业化作为开封快速崛起的基础支撑，稳定提高农业综合生产能力，大力发展现代农业，积极促进农业增效、农民增收。实施高标准粮田“百千万”建设工程。结合粮食核心区建设，按照“合理规划、综合配套、稳定面积、主攻单产”原则，规划建设一批百亩方、千亩方和万亩方高标准永久性粮田，统筹推进水、电、路、林等田间生产设施建设，力争全年粮食总产52亿斤以上。进一步抓好水利基础设施建设。抓好水利建设“2333”工程，即加快黑岗口、赵口2个调蓄水库建设，完善老城区、开封新区、郑汴新区3个水系规划建设，加大贾鲁河、惠济河、涡河3条河流的治理力度，加快赵口、柳园口、三义寨3个大型灌区续建配套与节水改造工程建设。切实抓好小型农田水利建设，做好“油改电”等田间工程，加快中低产田改造和土地治理。加快农业科技进步。大力推动农业科技创新，继续抓好“亩产800公斤超级小麦新品种培育与示范”等重大科技专项和重点科技计划项目的实施，加快小麦、玉米、水稻等粮食主导作物品种的换代升级。加强国家现代农业产业技术体系建设，深入推进花生、小麦、棉花、西甜瓜4个国家级综合试验站建设，加快农业科技成果转化和推广。加快推进基层农技推广服务体系建设，健全乡镇农业公共服务机构，积极推进生态农业和观光农业发展。大力发展现代农业。实施现代农业产业化集群培育工程，深入推进农业结构调整，重点打造粮食、油料、棉花、大蒜、畜禽、木业、花卉、蔬菜、淡水养殖、食用菌等10大产业链，建设粮食加工、棉纺加工、油脂加工、木材加工、畜禽加工、特色种植加工、香精香料、菊花产业等8大农业产业化集群。大力发展现代畜牧业，强化重大动（植）物疫病防控和农畜产品质量安全监管，全力打造优质畜产品供应基地。加强对农业产业化龙头企业服务，重点扶持天丰面粉、杞县大用、福甬纺织、中原皓月、雏鹰农牧等龙头企业，加快中原农副产品物流园区等农产品市场建设，培育年销售收入10亿元以上农产品加工企业3家以上，力争实现国家级农业产业化龙头企业零的突破。大力发展农民专业合作组织，新增农民专业合作社200家、农机专业合作社30家。抓好国家强农惠农富农政策落实。认真落实农资综合直补、良种补贴和农机具购置补贴等强农惠农富农政策；加大金融支农力度，积极发展村镇银行等新型农村金融机构；按照新的国家扶贫标准，积极推进扶贫开发，完成40个扶贫开发整村推进、5.3万贫困人口脱贫任务。加大农村劳动力技能培训力度，全年新增转移就业农村劳动力5万人。

（七）着力提高改革开放水平，为打好“五大攻坚战”提供强力支撑。打好“五大攻坚战”，改革是动力，开放是关键，要在更深层次深化改革，在更广阔领域扩大开放。继续深化企业改革。加快企业改革改制步伐，基本完成塑料厂、丝织印染厂依法破产；积极稳妥推进羊洋集团、空间结构厂改革；完成联运公司产权转让；做好矿山设备厂、磁性材料厂、一木公司等企业的破产重组工作。着力推进社会事业改革。继续深化事业单位人事制度改革，全面推行聘用制度、岗位管理制度和公开招聘制度。深入推进文化事业单位改革、医药卫生体制改革及其它领域改革。深化农村改革。坚持农村基本经营制度不动摇，认真搞好土地确权登记颁证。加强土地承包经营权流转管理和服务，发展适度规模经营。推进国有农场、林场体制改革。加大招商引资力度。围绕8大产业集聚区和十大产业集群，大力开展招商引资，力争新谋划亿元以上项目100个、新签约亿元以上项目100个、新开工亿元以上项目100个。把2012年作为“招商引资项目落实年”，切实做好签约项目跟踪落实工作，进一步提高合同履约率、资金到位率、项目开工率和达产率。创新招商引资方式。实施党政一把手招商引资工程，落实二分之一工作法，大力开展以商招商、驻地招商、区域招商、委托招商、对口招商、商会招商，不断提高招商引资、承接产业转移的针对性和实效性。办好第30届菊花花会经贸洽谈会等活动。改善投资环境。健全重大项目联审联批机制，提高项目审批效率。创新“涉企收费一费制”、“项目无偿代理制”等服务措施，完善外商权益保护长效机制，努力营造亲商、富商、安商的开放氛围。大力发展外向型经济。扩大工业产品进出口规模，加快“走出去”步伐，促进外向型经济发展平台建设。积极引导企业开展资源开发合作，促进外派劳务健康发展。

（八）着力转变发展方式，增强可持续发展能力。围绕转变发展方式这条主线，着力提高自主创新能力，加强环境保护和生态建设，推动节约集约发展。着力提高自主创新能力。加快国家级空分设备质检中心建设，提升空分设备工程技术研究中心水平，争创国家级工程技术研究中心。扎实推进国家级超级小麦育种国际合作基地建设，积极创建全国优秀“国际科技合作基地”；加快实施重大科技专项，加速科技成果转化，创建省级科技创新（成果转化）产业基地。高标准建设中国农业大学开封实验站。大力实施品牌战略，新培育国家级名牌产品2个，省级名牌产品6—10个。加强环境保护和生态建设。实行环境容量预算管理，推行排污权有偿使用和交易制度。启动“十二五”重点流域水污染防治工作，加大惠济河、贾鲁河等重点河流治理力度。加快实施黄汴河治污工程、雨污分流和污泥处理。进一步加大大气污染防治力度，加强饮用水源保护。建设新区污水处理厂和各产业集聚区污水处理厂，提升全市污水处理能力。加快垃圾处理场和生活垃圾发电项目建设。推进农村环境连片综合整治，加强农村污染治理，创建全省农村环境连片综合整治示范市。继续推进林业生态市建设，打造生态园林城市。推动节约集约发展。加快淘汰高耗能、高污染落后生产能力。积极推进节水技术改造。落实差别化土地管理政策，坚持最严格的耕地保护制度和最严格的节约用地制度，大幅度提高单位建设用地投资强度和单位土地面积产出率。

（九）切实保障和改善民生，促进社会和谐稳定。把保障和改善民生放在更加突出的位置，加大民生投入，办好一批实实在在的民生工程，着力解决一批人民群众最关心、最直接、最现实的利益问题，努力提高城乡居民幸福指数。千方百计扩大就业。加强就业岗位扩容、技能培训、信息服务和就业帮扶，支持小微型企业发展，重点做好高校毕业生、农村转移劳动力、城镇就业困难人员、退役军人等重点人群的创业就业工作，新增城镇就业5.8万人、再就业2.3万人。进一步完善社会保障体系。继续扩大社会保险覆盖面，实现城乡居民社会养老保险制度全覆盖。进一步完善被征地农民社会保障机制。完善城乡社会救助制度，不断提高企业退休人员、城乡低保、优抚对象和困难群众等各项社会保障标准，让人民群众得到更多实惠。积极推进社会福利园区建设。大力发展社会事业。继续加大教育投入力度，深化农村义务教育经费保障机制改革，推进义务教育均衡发展；深入实施学前教育三年行动计划，新改扩建幼儿园150所；适应城镇化需要优化中小学布局，加强薄弱学校改造。大力支持河南大学建设高水平大学，支持河南大学百年校庆活动，推动黄河水利职业技术学院、开封大学、开封文化艺术职业学院等高校加快发展。全面加强人才工作，大力培养造就创新创业人才、青年人才和急需紧缺人才，引进高层次人才。大力实施文化惠民工程，深入开展“舞台艺术送农民”、“高雅艺术进校园”、“农村电影放映工程”等活动。加强互联网管理，不断发展健康向上的网络文化。支持图书馆、文化馆建设，办好农家书屋。巩固城乡基本药物制度成果，持续推进基层医疗卫生机制改革和公立医院改革。新型农村合作医疗财政补助标准提高到240元，大病住院报销封顶线提高到15万元，参合率稳定在99%以上；加快医疗卫生资源整合，推进精神病医院迁建工作。着力解决进城务工人员的就业、安居、子女就学、社会保障等突出问题。统筹做好人口和计划生育工作，促进人口与经济社会协调发展。加强妇女儿童保护工作，维护残疾人的合法权益，积极发展老龄事业。开展全民健身活动，不断提升中国郑开国际马拉松赛水平。加强和创新社会管理。进一步加强和完善党委领导、政府负责、社会协同、公众参与的社会管理格局。建立科学有效的利益协调、诉求表达、矛盾调处和权益保障机制，维护群众合法权益。强化城乡社区自治和服务功能，探索新型社区管理和服务体制。大力推进政务诚信、商务诚信、社会诚信建设。加强食品药品监管，确保食品药品安全。加强市场监管和调控，确保生活用品价格基本稳定。加强对担保公司等金融业监管，着力营造良好金融环境。落实安全生产责任制，防止重特大安全事故发生。深化“平安开封”建设，强化社会治安综合治理，完善应急管理体制，提高各种风险的预防预警和应对能力。开展“六五”普法和法律援助工作。强化“双拥”共建，支持驻汴部队、民兵预备役和人防建设，促进军民融合式发展。全面落实党的民族宗教政策。继续做好外事、侨务、对台、统计、史志、气象、地震、社科研究等工作。

四、建设人民满意的服务型政府

加强政府自身建设，是全面完成今年各项目标任务的重要保证。必须坚持用领导方式转变加快发展方式转变，按照“三具两基一抓手”工作法，深入落实“两转两提”，求实求效，务实发展，不断提高执政能力和服务水平，持续提升政府公信力和执行力，建设人民满意的服务型政府。

（一）坚持为民行政。始终坚持把实现好、维护好、发展好最广大人民的根本利益，作为政府一切工作的出发点和落脚点，以高效、便民、负责为目标，坚持问政于民、问需于民、问计于民，把群众呼声作为第一信号，把群众愿望作为第一导向，把群众疾苦作为第一责任，牢固树立以人为本、执政为民理念，用为民的执政实践，创造更加丰硕的惠民成果，让开封人民生活得更加富裕、更加和谐、更加自信、更加幸福。

（二）坚持科学行政。要科学谋划，坚持一切从实际出发，准确把握影响开封发展的主要矛盾，按客观规律办事，增强工作谋划的科学性、系统性、前瞻性，不搞形象工程，不搞短期行为。要科学决策，把公众参与、专家论证、风险评估、合法性审查和集体讨论决定作为重大决策的必经程序，保证重大决策部署符合市情、顺应民意、不走错路、少走弯路。要科学运作，按照“三具两基一抓手”工作法，进一步转变工作作风，创新运行机制，优化工作流程，强化执行能力，提高工作效率。我们的工作实绩要经得起实践的检验，经得起人民的检验，经得起历史的检验。

（三）坚持民主行政。主动接受人大及常委会的法律监督、工作监督和政协的民主监督，认真办理人大代表建议和政协提案。广泛听取各民主党派、工商联和无党派人士的意见和建议，支持工会、共青团、妇联等人民团体发挥桥梁纽带作用。深化政务公开，重点推进财政预算、公共资源配置、重大项目建设审批等领域的信息公开，接受社会监督和媒体监督，让政府在监督中透明，让权力在阳光下运行。

（四）坚持依法行政。深入落实依法治国基本方略，全面推进依法行政，进一步加强法治政府建设。政府及部门都要严格在宪法和法律范围内活动，严格按照法定权限和程序行使权力、履行职责。规范行政执法行为，落实行政执法责任和过错追究制度，确保严格执法、公正执法、文明执法。加强法制宣传教育，努力营造有法必依、执法必严、违法必究的法制环境。

（五）坚持务实行政。要求真务实、真抓实干，以务实行政推动务实发展。要着力提高行政执行力，将战略谋划和决策部署落实到破解一个个发展难题上，落实到一个个具体项目上，明确到一个个责任主体上。对认准的事，要坚定执着，不动摇、不懈怠；对定下来的事，要雷厉风行，不推诿、不拖拉；对部署了的工作，要一抓到底，务求突破、务求实效。特别是对一些事关全局、事关长远、事关民生的大事、要事、急事、难事，要深入实际，直面困难，敢于担当，敢于破解，咬定青山不放松，不达目的不收兵。

（六）坚持廉洁行政。严格落实党风廉政建设责任制，完善工程建设、土地管理、国有资产转让、政府采购等制度，从源头上预防腐败。全面加强对重点领域、重点部门和重点资金的行政监察和审计监督，严查重点领域和关键岗位腐败。倡导勤俭干事业，力戒奢侈浪费，严格控制“三公经费”，降低行政成本。政府及部门工作人员都要做到警钟长鸣、秉公用权，坚守做人底线，立身不忘做人之本；坚守做事原则，用权不谋一己之私；坚守做官标准，为政不移公仆之心，以廉政建设的新成果保障发展，以清正廉洁的新形象取信于民。

各位代表！

中原经济区建设为国之大事、省之大计、民之大业，来之不易，一定要倍加珍惜；郑汴一体化发展战略深入人心、加速推进、作用凸显，来之不易，一定要倍加珍惜；打好“五大攻坚战”的序幕已经拉开，全市经济社会发展持续呈现好的趋势、好的态势、好的气势，来之不易，一定要倍加珍惜。

中原崛起，开封前程锦绣；河南振兴，古都机遇无限。让我们紧密团结在以胡锦涛同志为总书记的党中央周围，高举邓小平理论和“三个代表”重要思想伟大旗帜，深入落实科学发展观，以更加振奋的精神、更加开阔的视野、更加务实的作风，万众一心、奋发图强，精诚所至、金石开封，奋力开创我市经济社会发展新局面，为推动开封快速崛起而不懈奋斗，以优异成绩迎接党的十八大胜利召开！
